# Seismic Stability of Subsea Tunnels Subjected to Seepage

**DOI:** 10.1155/2014/631925

**Published:** 2014-03-18

**Authors:** Xuansheng Cheng, Yi Ren, Xiuli Du, Yida Zhang

**Affiliations:** ^1^School of Civil Engineering, Lanzhou University of Technology, Lanzhou 730050, China; ^2^Key Laboratory of Urban Security and Disaster Engineering of Education, Beijing University of Technology, Beijing 100124, China; ^3^Department of Civil and Environmental Engineering, Northwestern University, Evanston, IL 60208, USA

## Abstract

Strength reduction method and ADINA software are adopted to study the stability of submarine tunnel structures subjected to seepage and earthquake under different seawater depths and overlying rock strata thicknesses. First, the excess pore water pressure in the rock mass is eliminated through consolidation calculation. Second, dynamic time-history analysis is performed by inputting the seismic wave to obtain the maximum horizontal displacement at the model top. Finally, static analysis is conducted by inputting the gravity and the lateral border node horizontal displacement when the horizontal displacement is the largest on the top border. The safety factor of a subsea tunnel structure subjected to seepage and earthquake is obtained by continuously reducing the shear strength parameters until the calculation is not convergent. The results show that the plastic zone initially appears at a small scope on the arch feet close to the lining structure and at both sides of the vault. Moreover, the safety factor decreases with increasing seawater depth and overlying rock strata thickness. With increasing seawater depth and overlying rock strata thickness, maximum main stress, effective stress, and maximum displacement increase, whereas displacement amplitude slightly decreases.

## 1. Introduction

With the recent rapid development of global economy and engineering technology, tunnel construction has become increasingly important in regional economic and social development. In contrast to mountain tunnels, subsea tunnels have overlying unlimited seawater, thin rock layer, complex geological structure, and high risk of construction safety because of the particularity of the geological environment. Therefore, the seismic stability of tunnels should be investigated [1].

The stability of tunnels has always been a problematic aspect of tunnel design. Lee et al. [2] used tunnel stability limit analysis; however, limit analysis usually first supposes surface rupture and considers the stress state of a rock under plane failure. In recent years, finite element strength reduction has been widely used in slope and foundation projects. Zheng et al. [3–5] used this method to analyze the stability of the rock mass that surrounded tunnels by determining the safety factor of rock stability and calculating the position and shape of the failure surface of the rock mass that surrounded tunnels. Jiang et al. [6] discussed a calculation method for the overall safety factor of underground cavities that is based on strength reduction principle. Yang and Huang [7] analyzed the stability of shallow buried tunnels. Li et al. [8] considered the minimum safety factor in the stability analysis of the rock mass that surrounded tunnels. Li et al. [9] studied the static coupling effect of water and rock in relation to Xiamen undersea tunnel stability calculation and found that the effect of seepage is not negligible. Liu et al. [10] analyzed the dynamic response of shield tunnel under seismic load by using 2D dynamic finite element simulation. Li et al. [11] introduced strength reduction method into the security and stability analyses of reinforced concrete-immersed tunnels under static load. Yang et al. [12] analyzed the stability during excavation of shield tunnel. Akhlaghi and Nikkar [13] studied the seismic behavior of circular tunnels. Saito et al. [14] proposed that reducing rock permeability would change pore water pressure and prompt an expansion of the plastic zone of the rock mass that surrounded tunnels. Yin et al. [15] used finite element strength reduction method to analyze the stability of subsea tunnels. Many studies have also investigated the seismic stability of loess tunnels [16–20].

To date, the seismic stability of channel tunnels subjected to seepage remains unreported. The stability analysis of underwater tunnels is focused on tunnels across rivers, whose geological conditions are mostly saturated soft. By contrast, subsea tunnels have smaller coverage layer thickness, and most of the rock mass that surround these tunnels is fractured rock, with saturated weathered rock and breeze. Therefore, this study aims to determine the stability of submarine tunnels under seepage and seismic loading. The changes in shear strength safety coefficient are studied to provide theoretical basis for the calculation of the safety factor for subsea tunnels surrounded by rock mass and the engineering application of such factor during earthquakes. In this study, the influence of viscous elastic artificial boundary and seepage is considered, the finite element software ADINA is used, a tunnel fluid-solid interaction (FSI) model is established, and strength subtraction is performed.

## 2. Strength Reduction

The concept of shear strength reduction factor was first proposed in 1975 by Zienkiewicz et al. [21] in geotextile plastic finite element analysis. This method differs from the traditional method because the damaged surface is not considered. The shear strength reduction factor is obtained through the continuous reduction of the rock strength parameters, including cohesion *c* and internal friction factor *φ*, and through repeated calculation until the rock strength reaches a critical damage state. Elastic-plastic finite element analysis reveals that the program automatically obtains surface failure. From the actual state of rock to the failure state of that, the multiple of rock strength reduction is called as the strength reduction factor or the stability safety coefficient.

Let

(1) $$\begin{array}{c} c^{\prime }=\frac{c}{\eta },\;\;\varphi^{\prime }=\arctan\left(\frac{\tan\varphi }{\eta }\right). \end{array}$$

Then

(2) $$\begin{array}{c} \tau =\frac{c}{\eta }+\sigma \frac{\tan\varphi }{\eta }=c^{\prime }+\sigma \tan\varphi^{\prime }. \end{array}$$

## 3. Boundary Conditions and Seismic Waves

### 3.1. Viscoelastic Artificial Boundary

Deeks and Randolph [22] proposed the viscoelastic artificial boundary based on viscous boundary and used the artificial boundary formed by adding springs and dampers on the boundary. This boundary can simulate the radiation of scattering wave and the elastic recovery properties of foundation. Moreover, this boundary has good frequency stability and convergence displacement. The viscoelastic artificial boundary can be modeled as a continuous distribution of a parallel spring-damper system. The spring stiffness and damping values along the normal and tangential directions can be determined using the following formulas:

(3) $$\begin{array}{c} K_{BN}=\alpha_{N}\frac{G}{R},\;\;C_{BN}=\rho c_{p}, \end{array}$$

(4) $$\begin{array}{c} K_{BT}=\alpha_{T}\frac{G}{R},\;\;C_{BT}=\rho c_{s}, \end{array}$$

(5) $$\begin{array}{c} c_{p}=\sqrt{\frac{E\left(1-\mu \right)}{\rho \left(1+\mu \right)\left(1-2\mu \right)}},\;\;c_{s}=\sqrt{\frac{G}{\rho }}, \end{array}$$

where *K*_*BN*_ and *K*_*BT*_ are the normal and shear spring stiffness, respectively; *C*_*BN*_ and *C*_*BT*_ are the normal and shear damping coefficients, respectively;* R* is the distance from the wave source to the artificial boundary point; *c*_*p*_ is the wave velocity of the *P* wave; *c*_*s*_ is the wave velocity of the *S* wave; *G* is the shear modulus of the media; *ρ* is the mass density of the medium; *α*_*N*_ is the correction factor of the normal viscous boundary; *α*_*T*_ is the correction factor of the tangential viscous boundary (in general, *α*_*T*_ = 0.35 ~ 0.65 and *α*_*N*_ = 0.8 ~ 1.2. In this paper, *α*_*T*_ = 0.5 and *α*_*N*_ = 1.0); *E* is the elastic modulus; and *μ* is Poisson's ratio.

According to (3) and (4), *C*_*BN*_ is 1.2 × 10^7^ N/m, *K*_*BN*_ is 7.44 × 10^7^ N*·*S/m, *C*_*BT*_ is 7.58 × 10^6^ N/m, and *K*_*BT*_ is 3.72 × 10^7^ N*·*S/m. The spring element is used in ADINA, and the tangential and normal viscoelastic constraints are, respectively, imposed on both sides of the calculation model.

### 3.2. Input of Seismic Wave

**Figure 1:**
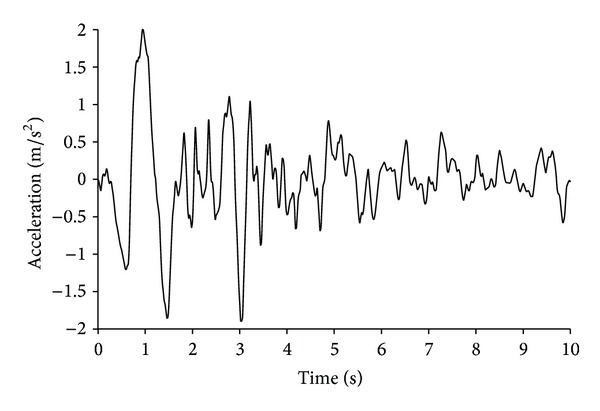
El-Centro seismic wave.

A seismic function should consider the depth of the seismic bedrock and the actual seismic waves on the surface of the bedrock. For simplicity, only the horizontal vibration of the seismic waves is considered. The underground antiseismic problem is very complicated. By contrast, seismic problems on the ground are well established, and various parameters can be measured and investigated. Therefore, seismic response is analyzed using the El-Centro SN wave by referring to the practice of the upper structure and inputting the seismic waves along the horizontal direction from the bottom boundary of the model. The duration of the wave is 10 s, and the peak of earthquake acceleration is approximately 1.96 m/s^2^ (intensity VIII) at 2 s. The peak is no longer adjusted, and the earthquake acceleration-time curve is shown in Figure 1.

## 4. FSI Calculation and Dynamic Finite Element Analysis

### 4.1. Tunnel Interaction Calculation under Seepage and Stress Fields

In the course of the fluid-solid coupling, the fluid affects the solid, and the deformation of the structure influences the fluid area. The mathematical model of coupling analysis of the seepage and stress fields can be expressed as follows [23]:

(6) $$\begin{array}{c} K_{p}\varphi +Q=S\frac{d\varphi }{dt},\\ \Delta \varepsilon_{v}=\frac{n\gamma }{E_{W}}\Delta \phi ,\\ \;\sigma =D\left(\varepsilon +\Delta \varepsilon_{v}\right), \end{array}$$

where **K**_*p*_ is the total seepage matrix; **Q** is the source phase array; **S** is the storage matrix; **σ** is the stress array of rock mass; **ε** is the strain matrix that does not consider osmotic pressure; Δ**ε**_*v*_ is the strain array of the rock deformation caused by permeable water pressure; and **D** is the elastic matrix. The stress field can be obtained from the interaction between the seepage and stress fields because the finite element method can solute the seepage field. Iterative method is used until the calculation satisfies the required precision. Finally, the permeability field and stress field distribution of the coupled analysis can be calculated as follows:

(7) $$\begin{array}{c} \varphi \left(x,y,z\right)=\frac{\varphi_{n}\left(x,y,z\right)+\varphi_{n+1}\left(x,y,z\right)}{2}\\ \sigma_{ij}\left(x,y,z\right)=\frac{\sigma_{ij}^{n}\left(x,y,z\right)+\sigma_{ij}^{n+1}\left(x,y,z\right)}{2}. \end{array}$$

Equation (7) is the numerical solution of tunnel coupling seepage field and stress field.

### 4.2. Dynamic Finite Element Analysis under Earthquake

The finite element matrix differential equation of the isolation body under earthquake action can be calculated as follows [16]:

(8) $$\begin{array}{c} M\ddot{u}\left(t\right)+C\dot{u}\left(t\right)+Ku\left(t\right)=-M{\ddot{u}}_{g}\left(t\right), \end{array}$$

where $\ddot{u}\left(t\right)$, $\dot{u}\left(t\right)$, and **u**(*t*) are the vectors of acceleration, velocity, and displacement of the isolation body node, respectively; **M**, **C**, and **K** are the matrix, damping matrix, and stiffness matrix of the isolation body, respectively; and ${\ddot{u}}_{g}\left(t\right)$ is the acceleration time history of the earthquake. Using Rayleigh damping, the damping matrix of isolated body is

(9) $$\begin{array}{c} C=\alpha M+\beta K, \end{array}$$

where *α* is the quality damping and *β* is the stiffness damping. *α* and *β* are calculated using the following formula:

(10) $$\begin{array}{c} \alpha =\frac{2\omega_{i}\omega_{j}}{\omega_{i}+\omega_{j}}\xi ,\;\;\beta =\frac{2}{\omega_{i}+\omega_{j}}\xi , \end{array}$$

where *ξ* is the damping ratio of *i* or *j* type vibration (*ξ*_*i*_ = *ξ*_*j*_ can also be obtained according to experimental data) and *ω*_*i*_ and *ω*_*j*_ are two different natural circular frequencies (through the modal analysis from isolation body). Equation (8) can be solved by using Newmark integral method

(11) $$\begin{array}{c} {\dot{u}}_{t+\Delta t}={\dot{u}}_{t}+\left(1-\gamma \right)t{\ddot{u}}_{t}+\gamma \Delta t{\ddot{u}}_{t+\Delta t}, \end{array}$$

where *γ* is a constant

(12) $$\begin{array}{c} {\ddot{u}}_{t+\Delta t}=\frac{4}{\Delta t^{2}}\left(u_{t+\Delta t}-u_{t}\right)-\frac{4}{\Delta t}{\dot{u}}_{t}-{\ddot{u}}_{t}, \end{array}$$

(13) u¨t(K+2ΔtC+4Δt2M)ut+Δt =C(2Δtut+u˙t)+M(2Δt2ut+4Δtu˙+u˙t)  −Mu¨g(t+Δt).

**u**_*t*+Δ*t*_ can be obtained using formula (13), whereas ${\ddot{u}}_{t+\Delta t}$ and ${\dot{u}}_{t+\Delta t}$ can be obtained using formulas (12) and (11).

The abovementioned analysis of the entire time-displacement process indicates that the moment *T*′ can be obtained at the maximum horizontal displacement of boundary vertices and that the horizontal displacement of the vertical boundary at *T*′ moment can be obtained.

### 4.3. Calculation Scheme

The influence of pore water pressure on rock stress and deformation must be considered to calculate the rock deformation containing groundwater. The rock pressure and pore water pressure acting on the tunnel lining stabilize after the completion of the tunnel. At this time, the excess pore water pressure no longer exists. The consolidation of undersea rock formations is simulated and the excess pore water pressure is eliminated from the rock to simulate the authenticity of the calculation results. When the horizontal displacement of model “vertices” is the largest on the right or left border, the moment *T*′ with maximum horizontal displacement of boundary vertice is obtained by adding seismic load, adopting a static analysis model, considering the gravity and the horizontal displacement of lateral border nodes. The safety factor of the tunnel structure under the actions of earthquake and seepage is obtained by continuously decreasing the strength parameters, including cohesion *c* and internal friction angle *φ*, until the calculation is not convergent. The calculation process is as follows: consolidation settlement calculation → changing boundary conditions and imposing seismic loading → restarting dynamic calculation → importing boundary displacement for reduction calculations.

## 5. Numerical Examples

### 5.1. Calculation Parameters

This study focuses on the tunnel structure stability analysis of ground motion under seepage. The rock is assumed to be homogeneous and isotropic. Moreover, it is regarded as the equivalent of continuous permeable media. The constitutive model of rock and concrete lining uses the Mohr-Coulomb material model. The seawater uses incompressible constant parameter model, with a unit of FCBI-C, a *ρ* of 10.09 kN/m^3^, and a bulk modulus of 10^20^ Pa. Relevant material parameters are shown in Table 1.

### 5.2. Calculation Model

**Figure 2:**
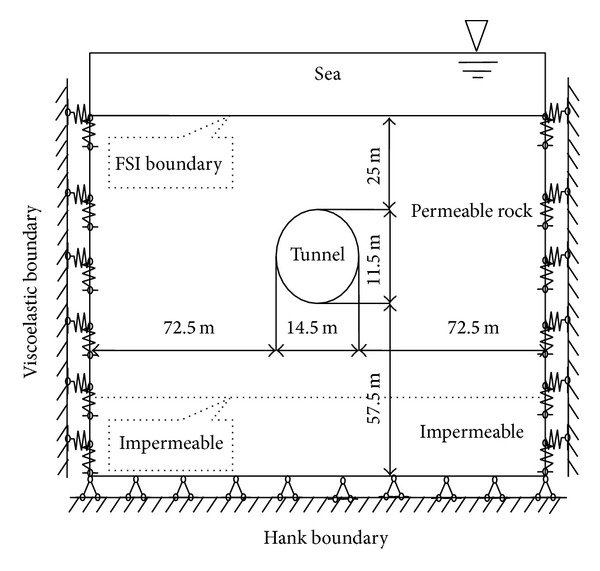
Analysis model.

Let the overlying water be 20, 30, or 35 m thick and let the tunnel overburden be 25, 35, or 45 m thick. The tunnel height is 11.5 m, and the span is 14.5 m. The thickness 1 from the semi-infinite body is cut because of the stability of rock mass. The calculation range from the bottom of the tunnel is five times that of the height, that is, 57.5 m. Each side of the tunnel is five times the tunnel span, that is, 72.5 m. Considering the influence of sea wave motion, we set the surface as free surface in ADINA. Considering the hydrodynamic pressure effect of seawater under earthquake, we define seawater and the contact surface of the overburden as FSI boundary. Considering the absorption ability of the viscoelastic boundary for the seismic wave, we set the surrounding rock displacement boundary as viscoelastic artificial boundary. The calculation model is shown in Figure 2, with a water depth of 20 m and an overburden thickness of 25 m.

### 5.3. Results and Analysis

#### 5.3.1. Influence of Different Water Depths on Stability

**Figure 3:**
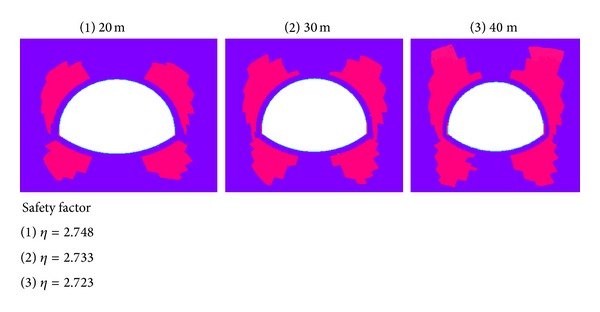
Plastic zones and safety factors with different water depths.

(*1)   Calculation of Safety Factor.* The tunnel overlying water depths are 20, 30, and 40 m, and the overburden thickness is 25 m. The safety factor and plastic zone distribution map for different water depths under seismic loading is shown in Figure 3.

As shown in Figure 3, the tunnel plastic zone first appears at small scope on the arch feet near the lining structure and at both sides around the vault. When the condition of the overburden thickness is constant, the water depth and the number of plastic areas are increased. Consequently, the safety factor is small and hardly changed.

(*2)   Results of Stress Calculation.* The stress on the subbottom tunnel, that is, the compressive stress, changes with the depth of the water.  Table 2 shows the maximum tension stress and maximum compressive stress of the tunnel when the thickness of the overlying strata is 25 m and the overlying water depths are 20, 30, and 40 m.

**Figure 4:**
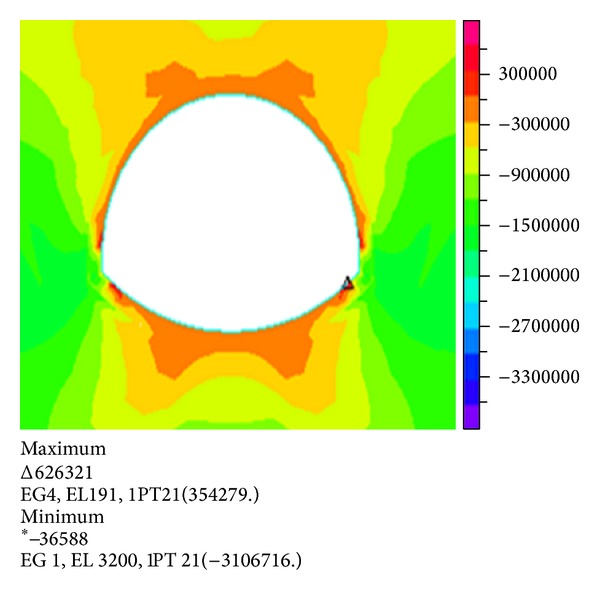
Nephogram of the first principal stress.

**Figure 5:**
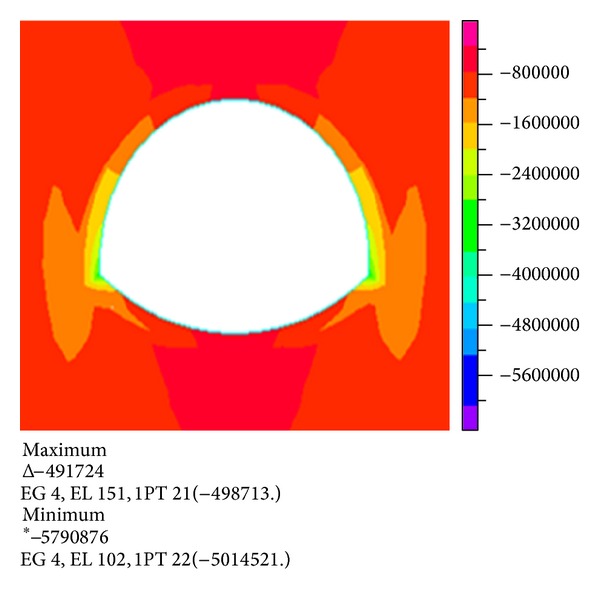
Nephogram of the third principal stress.

Figures 4 and 5 show the stress nephogram when the stratum thickness is 25 m and the depth of the overlying water is 20 m.

Comparison and analysis show that the maximum tensile stress and compressive stress gradually increase with increasing water depth under the same thickness of the overburden. The maximum tensile stress acts on the vault and inverted arch. The compression zone of the tunnel is mainly distributed on both sides of lining. Therefore, the lining of the arch feet should be specially strengthened. 

(*3)   Results of Displacement Calculation.* In consideration of the effects of seepage and earthquake, the shear strength parameters of the rock mass that surrounded the tunnel and the lining are continuously reduced until the calculation is not convergent. At this time, the maximum displacements along the *Y* direction (horizontal) and *Z* direction (vertical) of the tunnel structure under different water depths are shown in Table 3.

Therefore, with the same layer thickness, the maximum displacements along the *Y* and *Z* directions increase with increasing water depth. However, the growth rate decreases with increasing water depth. The displacements reach the maximum value at the same time, which indicates the overall vibration of the subsea tunnel, and the time variation of the horizontal and vertical displacement is the same.

### 5.4. Influence of Different Overburden Thicknesses on Stability

**Figure 6:**
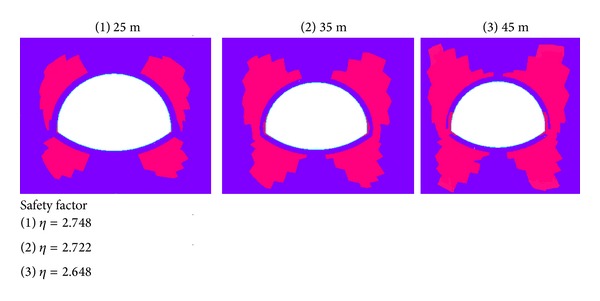
Plastic zones and safety factors with different thicknesses of the overburdens.

(*1)   Calculation of Safety Factor.* The tunnel overburden thicknesses are considered to be 25, 35, and 45 m, and the water depth is 20 m. The plastic zone maps and safety factors are shown in Figure 6.

As shown in Figure 6, the plastic zone first appears at small scope on the arch feet near the lining structure and at both sides of the vault. At constant water depth, a thicker overburden and more obvious plastic zone correspond to a smaller safety factor and gradual increase in variation amplitude.

(*2)   Results of Stress Calculation.* The stress on the structure, that is, compressive stress, changes with the thickness of the overlying strata.  Table 4 shows the maximum tension stress and maximum compressive stress of the tunnel when the depth of the overlying water is 20 m and the overburden thicknesses are 25, 35, and 45 m.

**Figure 7:**
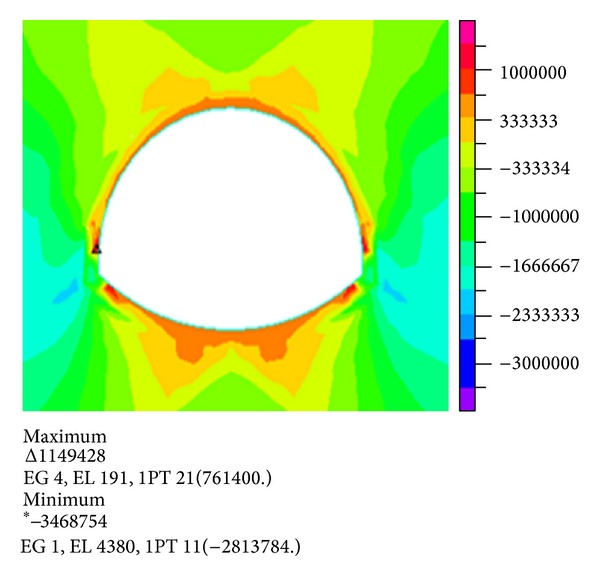
Nephogram of the first principal stress.

**Figure 8:**
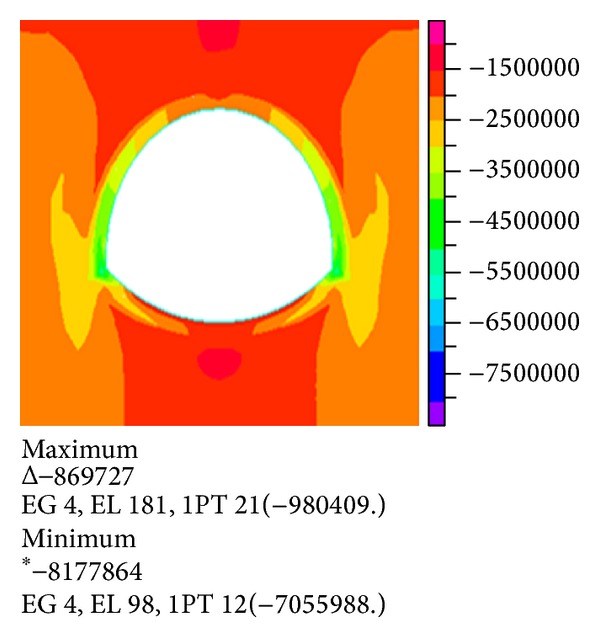
Nephogram of the third principal stress.

Figures 7 and 8 show the stress nephogram when the water depth is 20 m and the thickness of the overburden is 45 m.

Comparison and analysis show that the maximum tensile stress and compressive stress gradually increase with increasing overlying layer thickness under the same water depth. When the overlying rock increases to 45 m, the maximum principal stress and effective stress act on both sides of the arch foot.

The maximum tensile stress acts on vault and inverted arch. The compression zone of the tunnel is mainly distributed on both sides of lining. Therefore, the destruction of the pressure on the arch feet should be considered, and a number of appropriate measures should be adopted.

(*3)   Results of Displacement Calculation.* In consideration of the effects of seepage and earthquake, the shear strength parameters of the rock mass that surrounded the tunnel and the lining are continuously reduced until the calculation is not convergent. At this time, the maximum displacements of the *Y* direction (horizontal) and *Z* direction (vertical) of the tunnel structure for different overburden thicknesses are shown in Table 5.

Therefore, the maximum displacements of the *Y* and *Z* directions increase with increasing overburden thickness at the same water depth. The displacements reach the maximum value at the same time, which indicates the overall vibration of the subsea tunnel, and the time variation of the horizontal and vertical displacement is the same.

## 6. Conclusions

1. The plastic zone of subsea tunnel first appears at small scope on the arch feet near the lining structure and at both sides of the vault. Therefore, the structure should be strengthened because of the effects of earthquake.
2. The thicker the overburden layer is, the more obvious the plastic development is and the smaller the safety coefficient is, and the variation amplitude gradually increases.
3. The maximum principal stress increases when the thickness of the covering layer and the depth of the overlying seawater increase. The tensile area of subsea tunnel is mainly distributed in the vault and invert parts. The maximum tensile stress acts on the vault and inverted arch. The maximum compressive stress acts on both sides of lining. Therefore, the lining of the arch feet should be strengthened.
4. The maximum displacement increases with increasing water depth and layer thickness; however, the growth rate changes slowly with increasing depth.

## Figures and Tables

**Table 1:** Calculation parameters of materials.

Material name	Parameter							
	Modulus *E* (GPa)	Poisson's ratio *μ*	Specific weight *ρ* (kN/m^3^)	Cohesion *c* (KPa)	Internal friction angle *φ* (°)	Porosity *n* (%)	Permeability *K* (m/s)	Tensile strength *σ* (MPa)
Porous media	20	0.33	26	500	50	0.75	1.00*E* − 06	0.15

Material name	Parameter							
	Modulus *E* (GPa)	Poisson's ratio *μ*	Specific weight *ρ* (kN/m^3^)	Cohesion *c* (KPa)	Internal friction angle *φ* (°)	Porosity *n* (%)	Permeability *K* (m/s)	Tensile strength *σ* (MPa)

Nonporous media	20	0.35	26	500	50	—	—	0.15

Material name	Parameter							
	Modulus *E* (GPa)	Poisson's ratio *μ*	Specific weight *ρ* (kN/m^3^)	Cohesion *c* (KPa)	Internal friction angle *φ* (°)	Porosity *n* (%)	Permeability *K* (m/s)	Tensile strength *σ* (MPa)

Lining structure	30	0.167	24.5	3180	54.9	—	—	2.01

**Table 2:** Maximum principal stress under different water depths (MPa).

Water depth	20 m	30 m	40 m
Maximum tensile stress	0.626	0.834	0.911
Maximum compressive stress	5.791	6.336	6.392

**Table 3:** Maximum displacements of tunnel structure under different water depths.

Maximum displacement (mm)	Overlying water depth		
	20 m	30 m	35 m
*Y* ^max⁡^	−0.154	−0.180	−0.199
*Z* ^max⁡^	−4.382	−4.731	−4.883

**Table 4:** Maximum principal stress under different thicknesses of the overburden (MPa).

Thicknesses of the overburden	25 m	35 m	45 m
Maximum tensile stress	0.626	1.074	1.149
Maximum compressive stress	5.791	6.869	8.178

**Table 5:** Maximum displacements of tunnel structure under different overburden thicknesses.

Maximum displacement	Overburden thickness		
	25 m	35 m	45 m
*Y* ^max⁡^	−0.154	−0.247	−0.343
*Z* ^max⁡^	−4.382	−5.169	−5.930
